# Phospho-regulated tethering of focal adhesion kinase to vinculin links force transduction to focal adhesion signaling

**DOI:** 10.1186/s12964-025-02201-3

**Published:** 2025-04-21

**Authors:** Karen Diaz-Palacios, Pilar López Navajas, Bárbara Rodrigo Martín, Ruth Matesanz, Juan R. Luque-Ortega, Asier Echarri, Daniel Lietha

**Affiliations:** 1 Molecular and Cellular Biosciences, Margarita Salas Center for Biological Research (CIB), Spanish National Research Council (CSIC), Madrid, Spain; 2 Biomedicine, Margarita Salas Center for Biological Research (CIB), Spanish National Research Council (CSIC), Madrid, Spain; 3 Molecular Interactions Facility, Margarita Salas Center for Biological Research (CIB), Spanish National Research Council (CSIC), Madrid, Spain

## Abstract

Focal Adhesion Kinase (FAK) is a key signaling molecule in focal adhesions (FAs) orchestrating the formation, maturation and turnover of the FA complex. A controlled FA lifecycle is essential for various cellular processes requiring mesenchymal cell migration and is harnessed by advanced cancers to initiate cancer invasion and metastasis. The mechanical force for migration is transmitted from actin stress fibers to FAs via specialized force transduction components in FAs. These forces are known to activate FA signaling, suggesting a communication between FA force transduction and FA signaling components, yet how this occurs mechanistically is unknown. Here we demonstrate that paxillin can act as an adaptor protein to connect FAK with the force transduction component vinculin. Our data show that this connection forms inefficient in the basal state but suggest Y925 phosphorylation in FAK as a key mechanism for optimal formation of the FAK:paxillin:vinculin linkage. This is achieved by switching binding of the paxillin LD2 motif from FAK to vinculin while keeping paxillin LD4 tethered to FAK. We further provide the first high-resolution crystal structure of LD2 bound to the vinculin tail domain, which importantly shows that vinculin can simultaneously link to actin. This therefore ensures an intact force transduction role of vinculin while tethered via paxillin to the signaling apparatus in FAs. With this data, all interactions of the force transmitting tether to FAK are structurally defined and we provide an atomic model for FAK force activation. In summary, we propose a phospho-regulated connection between signaling and force transduction components in FAs allowing for force induced activation of FA signaling.

**Supplementary Information:**

The online version contains supplementary material available at 10.1186/s12964-025-02201-3.

## Introduction

Focal adhesions (FAs) are large complexes in the cell that form at the cytoplasmic side of integrin trans-membrane receptors [9, 53]. Extracellularly, integrins attach to extracellular matrix (ECM) proteins and intracellularly, connect via the FA complex to actin stress fibers. Forces generated by contracting stress fibers are transmitted via the FA complex and integrin receptors to the ECM to produce traction for cell migration [27]. For productive and directional migration this process needs to be tightly regulated in order to ensure stabilization of new (nascent) FAs at the cell front and disassembly of FAs at the rear of the cell. Coordination of these events in correct succession is ensured by a signaling apparatus integrated in the FA complex [54]. A central signaling component in FAs is Focal Adhesion Kinase (FAK) [34]. FAK is a non-receptor tyrosine kinase which is essential for efficient cell migration. FAK knock out cells exhibit excessive numbers of FAs that poorly turn over, inhibiting cell migration [23]. FAK is frequently overexpressed in various advanced solid tumors, including in lung, ovarian or breast cancer, and is associated with poor patient outcome [45, 56]. FAK is activated with increasing forces in FAs as occurs on stiff substrates [47, 50, 51, 57]. This mechanism is utilized by fibrotic tumors to drive tumor invasion via increased FA signaling [35].

**Fig. 1:**
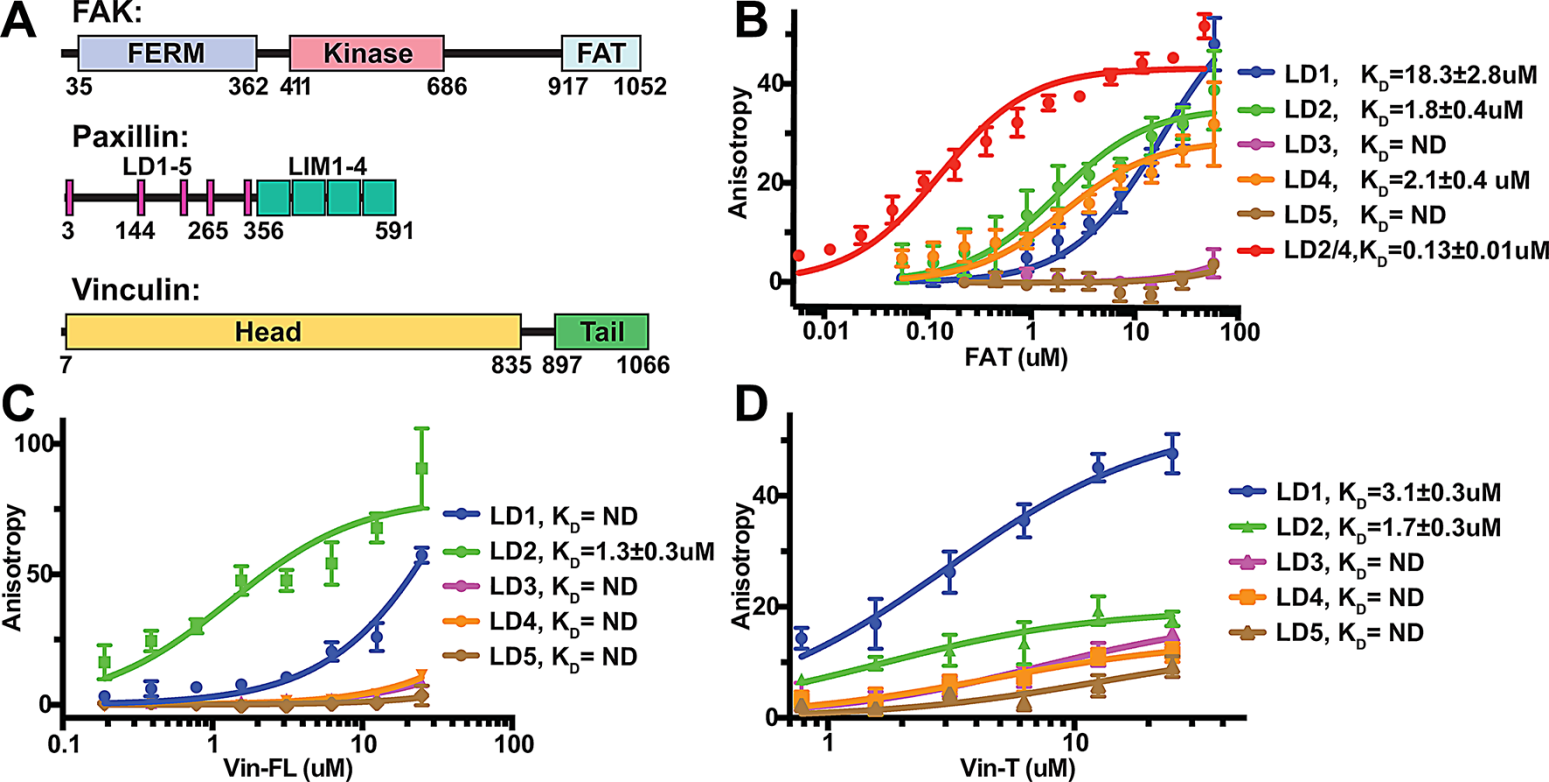
Binding specificity of paxillin LD motifs for FAK and vinculin. **(A)** Schematic domain structures of FAK, paxillin and vinculin. Residue numbers of domain boundaries are indicated; for LD motifs starting residue numbers for LD1, LD2 and LD4 are shown. **(B)** Binding curves for FITC labelled paxillin LD peptides to the FAT domain of FAK were determined by fluorescence anisotropy. LD2/4 contains sequences of LD2 and LD4 separated by a linker (see Tab.S1). **(C)** Binding curves for FITC labelled paxillin LD peptides to full-length vinculin (Vin-FL). **(D)** Binding curves for FITC labelled paxillin LD peptides to the vinculin tail domain (Vin-T). **(B-C)** Apparent K_D_ values are obtained from fitting a one-site binding model. Resulting K_D_ values with an error > 25% are considered not determined (ND). Error bars represent SD from 4 measurements

FAK contains a N-terminal FERM (four point one, ezrin, radixin, moesin homology) domain, a central kinase domain and a C-terminal focal adhesion targeting (FAT) domain (Fig. 1A). The FERM domain is responsible for FAK autoinhibition prior to FA localization [36], while the FAT domain recruits FAK to FAs. Once localized to FAs, the FERM domain interacts with phosphatidylinositol-4,5-bisphoaphate (PI(4,5)P2) lipids in the membrane, leading to rupture of autoinhibitory FERM: kinase interactions [7, 18]. This frees the kinase domain to reorient itself and also interact with PI(4,5)P2, resulting in a conformation where the kinase active site faces the membrane [2]. In this membrane bound state FAK forms an oligomeric assembly that promotes efficient trans-autophosphorylation of Y397 in the linker between FERM and kinase domains, but keeps turnover activity low due to active site occlusion by the membrane. Full activation of FAK occurs during FA maturation when forces build up in FAs and several tyrosines in FAK are phosphorylated by the Src kinase, including Y576 and Y577 in the FAK activation loop, Y861 in the kinase/FAT linker and Y925 within the C-terminal FAT domain [34].

Ultrastructural analysis of FAs has revealed a layered architecture, with a FAK containing signaling layer on the membrane, an actin-rich layer some 50 nm above the membrane and, connecting the two, an intermediate force transduction layer [28]. Two of the main force transduction components are talin and vinculin. Talin spans across FAs with its N-terminal head domain interacting with cytoplasmic integrin tails at the membrane and its C-terminus interacting with actin [16, 17]. Vinculin is initially recruited close to the membrane where it interacts with PI(4,5)P2 [10, 41]. As forces increase in FAs, vinculin moves away from the membrane towards the force transduction and actin rich layers in a myosin II dependent manner [8]. Both, talin and vinculin are required to maintain a dependency between the force observed in individual FAs and FAK activation [57], suggesting a role for these force transduction components in force-mediated activation of FAK. The FAT domain in FAK is shown to directly interact with the talin head domain and this interaction in thought to promote talin recruitment to nascent adhesions [33]. A direct interaction between FAK and vinculin is not reported, but both are shown to interact with the FA adaptor protein paxillin [5, 21].

With vinculin observed to move from the membrane towards actin in a force dependent manner [8], we hypothesize that a FAT:paxillin:vinculin linkage could transmit force to FAK to induce activation. Here we perform systematic reconstitution experiments and show that such a tripartite linkage forms. We demonstrate that it is promoted by release of the paxillin LD2 binding motif from FAT, allowing it to interact with vinculin, while the paxillin binding motif LD4 stays bound to FAT, hence establishing a paxillin tether linking FAK with vinculin. We further show that LD2 release from FAT is promoted by FAT phosphorylation on Y925, suggesting a phosphoregulated switch to establish the link. We show in cells that Y925 phosphorylation and maintaining the paxillin LD4 linkage to FAT is required for efficient ECM mediated FAK activation. We further provide new structural data for the LD2 interaction with the vinculin tail (Vin-T) domain, which reveals that Vin-T can simultaneously interact with LD2 and actin as required for force transduction via a vinculin:paxillin:FAK linkage. The structure provides the missing data to generate a full reconstruction of FAK tethering to force and we provide a complete atomic model that integrates the FAK-actin linkage into force transduction to integrins.

## Results

### Binary interactions in the FAT:paxillin:vinculin linkage

To test the potential connection of FAK to force transduction components in focal adhesions via a FAT:paxillin:vinculin linkage, we first analyzed the binary FAT:paxillin and paxillin:vinculin interactions using fluorescence anisotropy experiments. For this we used purified FAT and vinculin proteins with synthetic FITC labelled peptides corresponding to the five LD motifs in paxillin (LD1-LD5) (Tab.S1), previously implicated in FAK and vinculin interactions [21, 49]. In agreement with previous findings, we found that FAT binds with highest affinity to LD2 and LD4 (K_D_ ~2µM; Fig. 1B). In addition, we detected an approximately 10-fold lower affinity for LD1 and no significant affinity for LD3 and LD5. Using full-length vinculin (Vin-FL) we observed clear selectivity for LD2 (K_D_=1.3 ± 0.3µM) (Fig. 1C). Using the Vin-T domain, which was previously reported to mediate paxillin interactions [52], we detected binding for LD1 (K_D_=3.1 ± 0.3µM) and LD2 (K_D_=1.7 ± 0.3µM) (Fig. 1D). Notably, LD2 displayed low anisotropy signals, presumably due to FITC retaining significant mobility upon binding. Taken together, we confirmed selective binding of FAT to paxillin LD2 and LD4 and for Vin-T to LD1 and LD2.

**Fig. 2:**
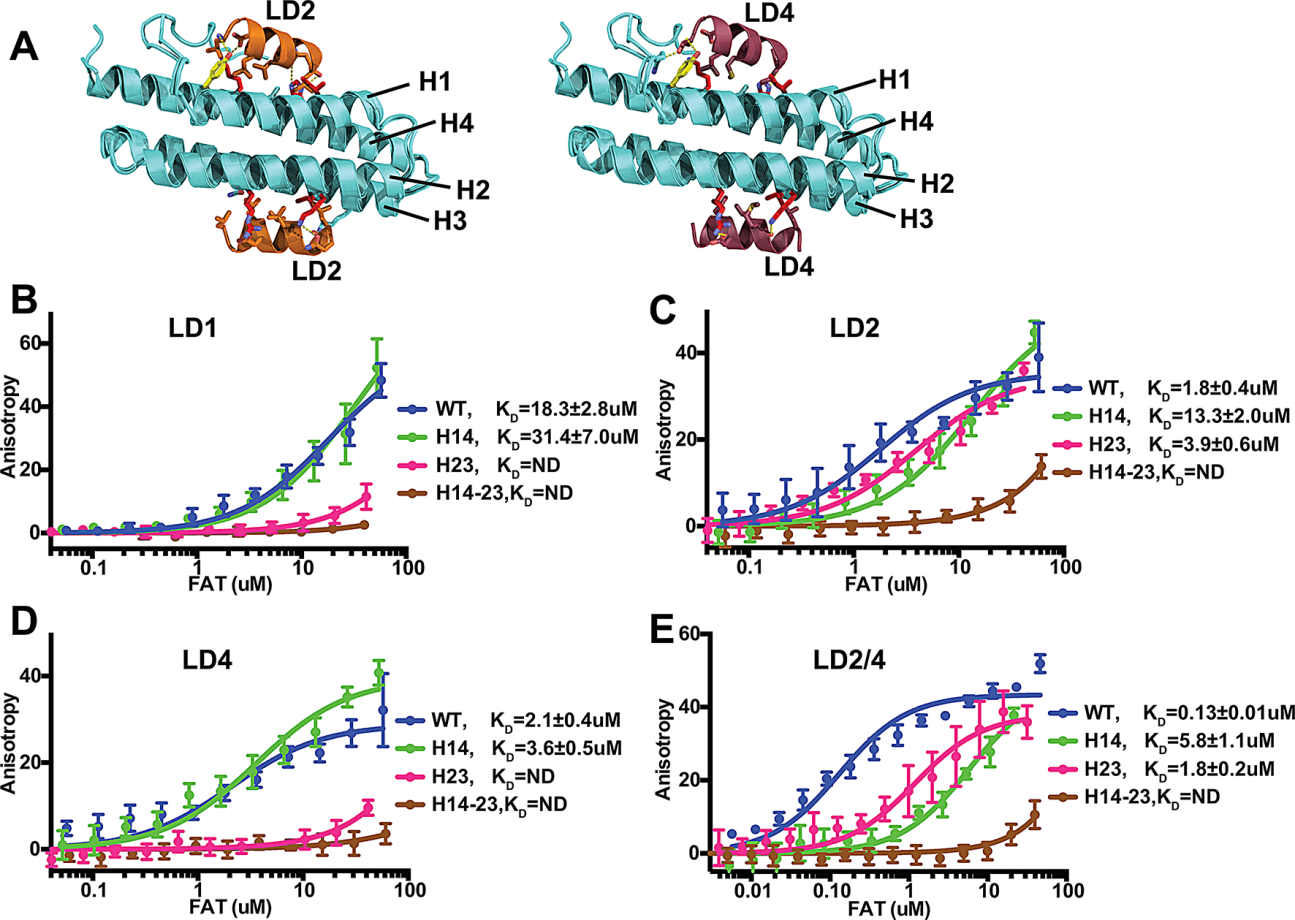
Binding specificity of paxillin LD motifs for the two FAT binding sites. **(A)** Crystal structures of paxillin LD2 and LD4 peptides bound to FAT [21]. At high peptide concentrations used for crystallization both LD motifs can bind FAT via two binding sites, one involving helices H1 and H4 (H14), the other via H2 and H3 (H23). Due to crystal packing the two sites are occupied on different FAT molecules, which are superimposed here. Side chains of residues mutated in H14 and H23 are colored red; the side chain of Y925 is colored yellow. **(B-E)** Binding curves for peptides LD1 (B), LD2 (C), LD4 (D) and LD2/4 (E) to FAT-WT or FAT mutated at LD binding sites H14 or H23 or both (H14-23). Apparent K_D_ values are obtained from fitting a one-site binding model. Resulting K_D_ values with an error > 25% are considered not determined (ND). Error bars represent SD from 4 measurements

Since FAT can interact with two LD motifs (LD2 and LD4) and previous crystal structures indicated the presence of two LD binding sites on opposite sides of the FAT four helix bundle (Fig. 2A) [21], we next measured the affinity of a peptide containing both, the LD2 and LD4 motifs (LD2/4; Tab.S1). In this peptide, the two LD motifs are separated by a linker designed to allow simultaneous binding of the two LD motifs to opposite faces of FAT. Using molecular dynamics simulations we observe similar binding behavior for this peptide as for the native LD2-LD3-LD4 region of paxillin (Fig.S1). Affinity measurement with the LD2/4 peptide revealed that the presence of both LD motifs synergistically increases the affinity of LD2/4 approximately 15-fold, resulting in a K_D_ of 0.13 ± 0.01µM (Fig. 1B).

In order to determine whether the paxillin peptides display selectivity for the two binding sites in FAT, we generated mutant forms of FAT with mutations, either in the binding site situated on helices 1 and 4 (H14), or in the binding site on helices 2 and 3 (H23), or in both (H14-23) (Fig. 2A). Binding assays with these mutant forms of FAT showed that the modest binding of LD1 is selective for site H23 on FAT, since its mutation strongly reduces affinity of LD1 (Fig. 2B). LD2 is less selective, but H14 mutations reduced the affinity ~ 7 fold, suggesting preferential binding of LD2 to the H14 site (Fig. 2C). Mutation of the H23 site had a modest effect, while mutation of both sites strongly reduced binding to residual levels. As LD1, LD4 binding is highly selective for the H23 site and the significantly higher affinity compared to LD1 suggests LD4 as the relevant binder for the H23 site in FAT (Fig. 2D). Binding of the LD2/4 peptide to both sites in FAT is confirmed by reduction of the affinity to values similar to single site binding for FAT mutants H14 and H23 and no significant binding to the double site mutant H14-23 (Fig. 2E).

### FAK, paxillin and vinculin form a tripartite complex

**Fig. 3:**
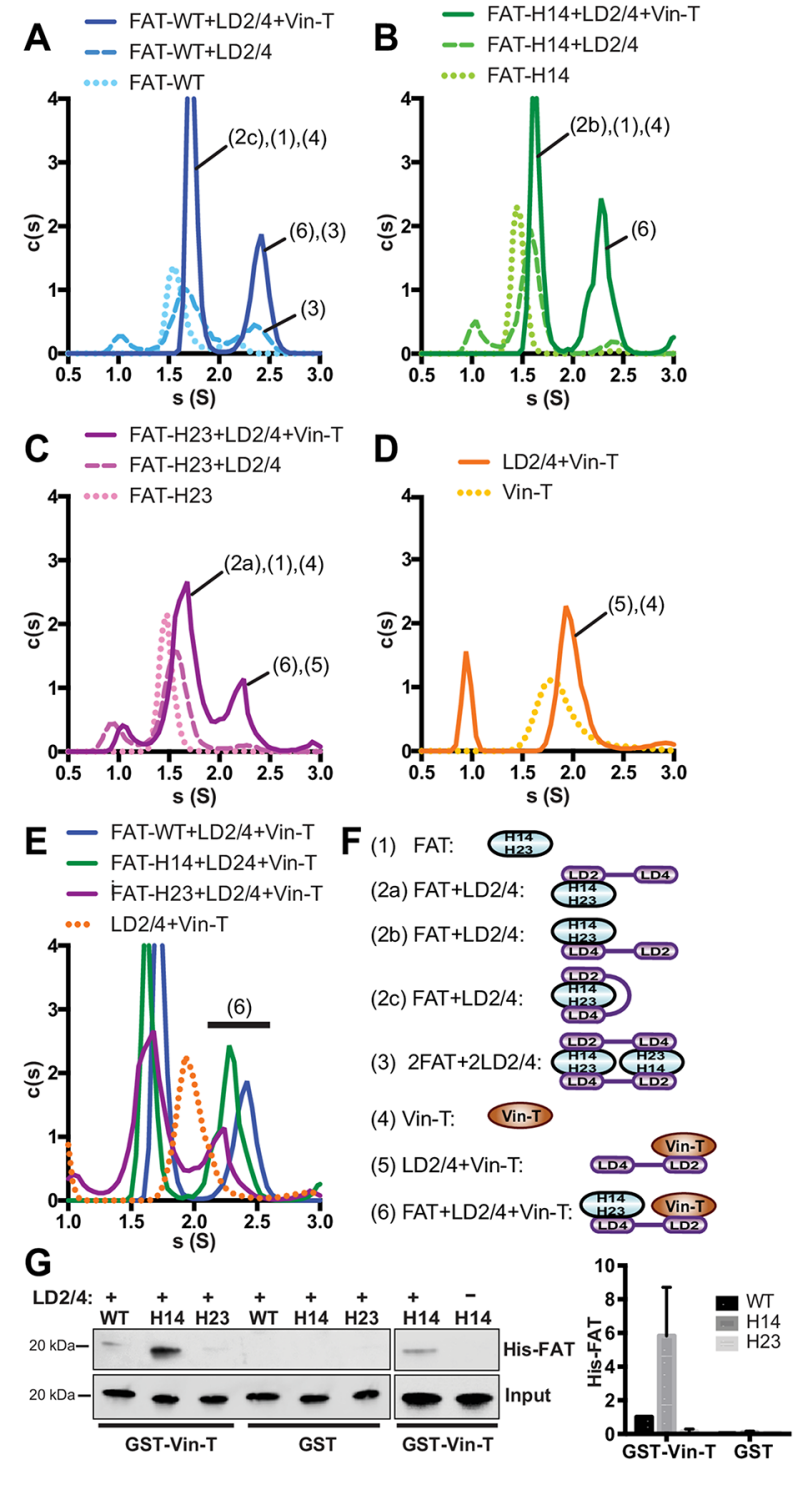
Formation of FAT:LD2/4:vinculin tail (Vin-T) ternary complexes observed by sedimentation velocity analytical ultracentrifugation (svAUC). **(A-D)** Continuous sedimentation coefficient (S) distribution curves are shown for samples indicated in the legends. Peaks are labeled with likely species they contain based on their size. Labelling is according to the key in panel (F). **(E)** Superposition of S distribution curves from samples containing the tripartite FAT:LD2/4:Vin-T ternary complex (species 6 in panel F) together with the LD2/4 + Vin-T sample (orange dotted curve). Note that compared to FAT-WT, ternary complex formation is increased for FAT-H14 and decreased for FAT-H23 and only for the latter the LD2/4:Vin-T binary complex (species 5 in panel F) remains detectable. **(F)** List of complexes and free proteins species potentially present in svAUC samples together with their schematic illustration and key used in panels A-E. **(G)** GST-pulldown experiments were performed with GST-fused Vin-T, LD2/4 and His-tagged FAT to confirm ternary complex formation. In agreement with AUC experiments, complex formation resulting in detection of His-FAT is promoted by H14 and reduced by H23 mutations. His-FAT-H14 is only pulled down in the presence of the LD2/4 peptide. A representative experiment is shown in the left panel and the averaged quantification from 3 experiments is plotted in the right panel. For quantifications signals were normalized to FAT-WT pulled down with GST-Vin-T. Error bars represent SEM from three independent experiments

Since both, the FAT domain of FAK and vinculin interact with paxillin (Fig. 1), we next tested whether the three components can form a tripartite FAK:paxillin:vinculin complex. For this we mixed FAT (30µM), Vin-T (30µM) and sub-stoichiometric amounts of the paxillin LD2/4 peptide (10 µM; in order to minimize binary paxillin complexes) and monitored complex formation by sedimentation velocity analytical ultracentrifugation (svAUC). Using FAT-WT we observed significant amounts of a species with a sedimentation coefficient of ~ 2.4 S, compatible with the tripartite FAT:LD2/4:Vin-T complex (Fig. 3A). This peak is also compatible with a 2:2 complex of FAT:LD2/4 (schematically shown as species (3) in Fig. 3F) and formation of this species is indeed suggested by a peak at ~ 2.4 S for a sample that only contains FAT-WT and LD2/4 (Fig. 3A). However, integration of the 2.4 S peaks in the two samples results in significantly lower values in absence (c = 0.15) compared to in presence of Vin-T (c = 0.40), suggesting formation of considerable amounts (c≈0.25) of the tripartite complex (for integrations of all peaks, see Tab.S2). Binary interaction studies (Fig. 1) suggest that the tripartite complex forms via LD2 binding to Vin-T and LD4 interacting with FAT (schematically shown as species (6) in Fig. 3F). Since LD2 can also interact with FAT, formation of the tripartite complex is likely not optimal for FAT-WT. We hypothesized that mutation of the H14 site in FAT (proposed as LD2 binding site) should promote, and mutation of the H23 site (proposed as LD4 binding site) should reduce formation of the tripartite FAT:LD2/4:Vin-T complex. Indeed, svAUC experiments resulted in an increase of the 2.4 S peak for FAT-H14 (c = 0.52) and a decrease for FAT-H23 (c≈0.30) (Figs. 3B-C), supporting our hypothesis. Notably, FAT-H14 does not form significant amounts of a 2:2 FAT:LD2/4 complex, hence the 2.4 S peak can be almost exclusively attributed to the tripartite complex. On the other hand, for FAT-H23, we observed a slight left shift and significant absorbance at ~ 2 S connecting the ~ 2.3 S and ~ 1.5 S peaks in the FAT-H23 + LD2/4 + Vin-T sample. This suggests the presence of significant amounts of the binary LD2/4:Vin-T complex, which although not resolved likely causes the left shift and significantly contributes to the 2.3 S peak (Figs. 3D-E). To further confirm formation of the ternary FAT:LD2/4:Vin-T complex, we performed GST pulldown experiments with GST fused Vin-T, LD2/4 and His-tagged FAT, detecting bound FAT via western blot and detection with an anti-His antibody. We confirmed that FAT-H14 forms a complex with GST-Vin-T only in the presence of LD2/4 (confirming ternary complex formation) and that the complex forms most efficiently with the FAT-H14 mutant (Fig. 3G). In conclusion, these results suggest that paxillin can tether FAK to vinculin, via interactions of LD2 with Vin-T and LD4 with FAT and this tripartite complex is promoted by disfavoring LD2 binding to the H14 site in FAT.

**Fig. 4:**
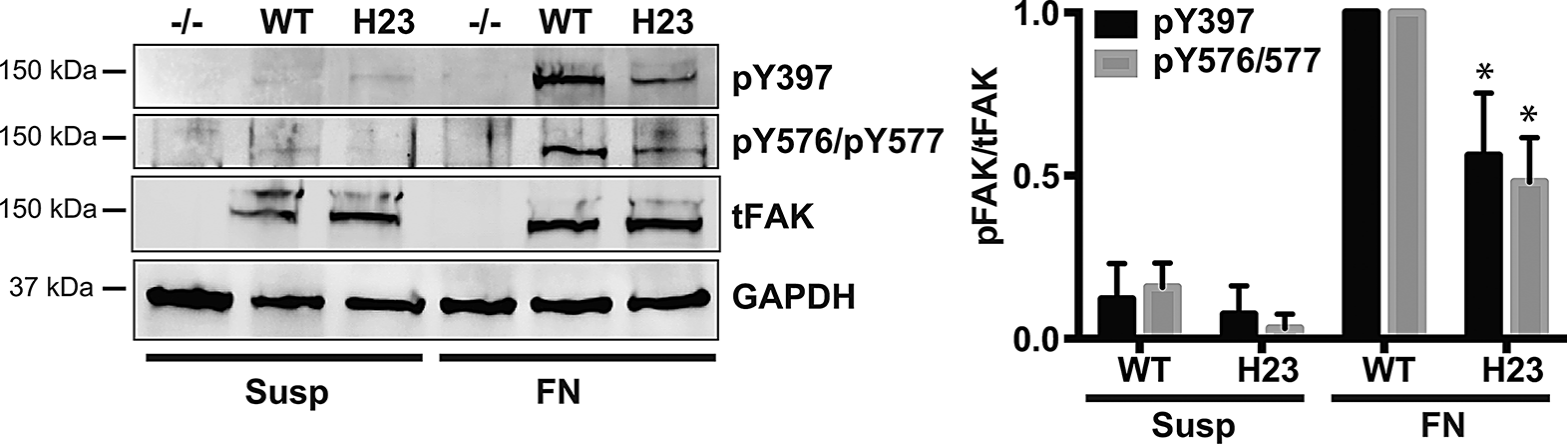
ECM mediated FAK activation in cells requires linkage of paxillin LD4 to the FAT-H23 site. FAK-WT or FAK mutated in the FAT H23 paxillin binding site were stably expressed into FAK-/- MEFs. ECM mediated FAK activation was induced by keeping cells in suspension (Susp) prior to plating them on fibronectin (FN). FAK activation was monitored by western blot using two phosphospecific antibodies, one to detect FAK autophosphorylation on Y397 and one for phosphorylation of FAK activation loop residues Y576 and Y577. A representative experiment is shown in the left panel and the quantification is plotted in the right panel. For quantifications signals were normalized to FAK-WT in MEFs on FN. Error bars represent SD from three independent experiments. *p = 0.02 (unpaired Student t test)

We next tested whether the FAT linkage to paxillin LD4 in the ternary complex is important for ECM mediated FAK activation in cells. For this, we stably expressed FAK with mutations in the FAT LD4 binding site (FAK-H23) or the FAK wild-type control (FAK-WT) in FAK deficient mouse embryonic fibroblasts (FAK -/- MEFs) and determined FAK activation after plating these cells on fibronectin. We monitored Y397 autophosphorylation and phosphorylation of the kinase activation loop residues Y576 and Y577 by western blot. As expected, in FAK-/- deficient cells and in cells kept in suspension, no phosphorylation signals were observed (Fig. 4). Importantly, upon cell adhesion to fibronectin FAK-H23 expressing cells exhibited significantly reduced phosphorylation levels compared to FAK-WT expressing cells, indicating inefficient FAK activation in absence of a FAT-paxillin LD4 linkage. Taken together, biochemical and cellular results support a model where FAK linkage via paxillin to vinculin is important for ECM mediated FAK activation.

### Y925 phosphorylation in FAT releases paxillin LD2 and is required for FAK activation

**Fig. 5:**
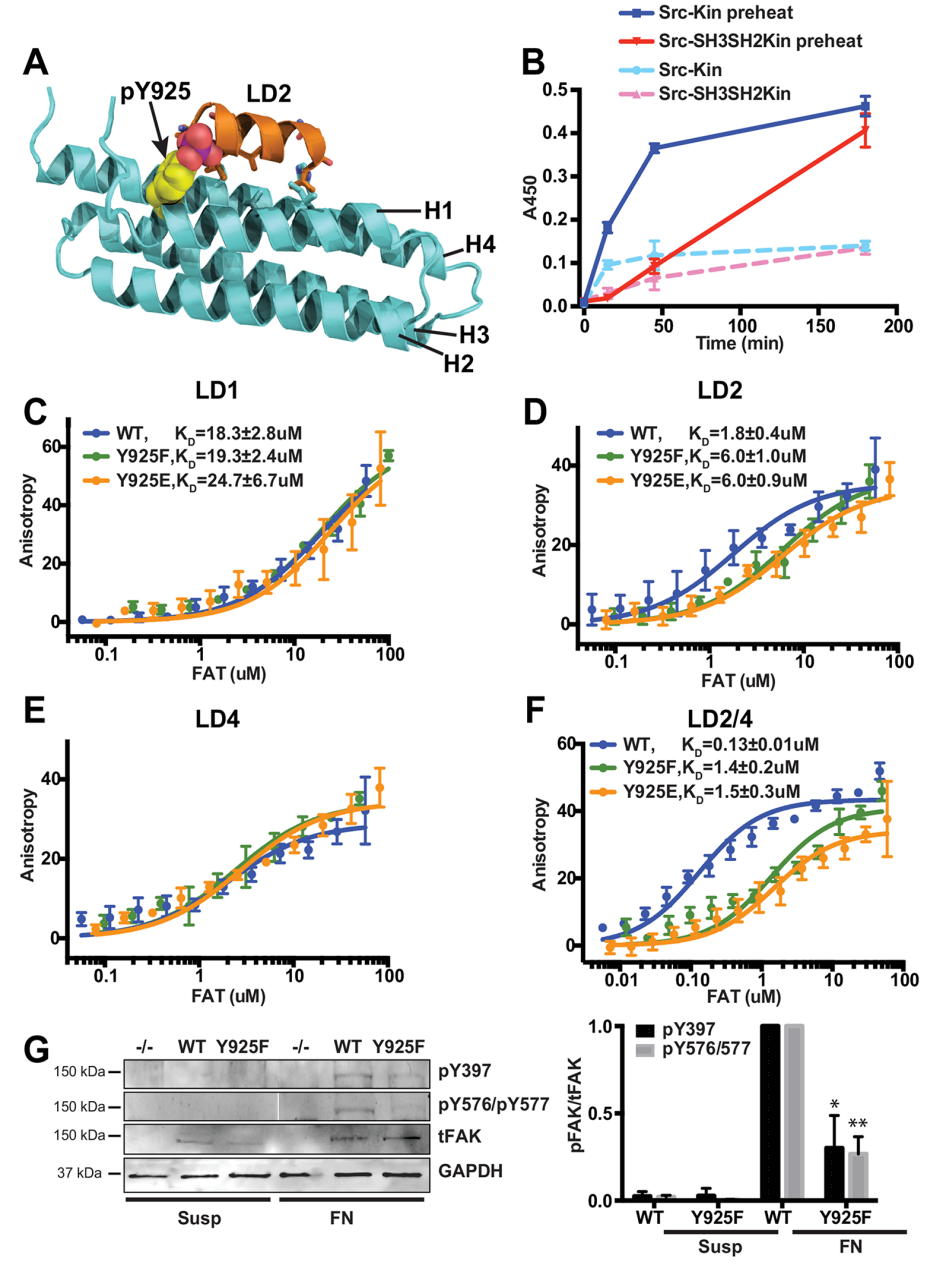
FAT phosphorylation affects LD2 binding to the H14 site in FAT. **(A)** Model of FAT phosphorylated on Y925 (pY925) with LD2 bound to the H14 site. pY925 is shown in space fill with carbons in yellow. The modelling suggests that Y925 phosphorylation impedes LD2 binding. **(B)** Time course of FAT phosphorylation on Y925 by Src, monitored by ELISA using a phosphospecific anti-FAK pY925 antibody. Assays were performed with the Src kinase domain (Src-Kin; residues 254–536) or Src SH3-SH2-kinase (Src-SH3SH2Kin; residues 84–536); without or with preheating the FAT domain to 95 °C prior to addition of Src. Error bars represent SD from 4 measurements (2 independent reactions). **(C-F)** Binding curves for peptides LD1 (C), LD2 (D), LD4 (E) and LD2/4 (F) to FAT-WT, phospho-deficient FAT-Y925F and phosphomimetic FAT-Y925E. Apparent K_D_ values are obtained from fitting a one-site binding model. Both Y925 mutations affect binding of LD2 and LD2/4, but not LD1 and LD4. Error bars represent SD from 4 measurements. **(G)** ECM mediated FAK activation in cells requires Y925 phosphorylation. FAK-WT or the phosphodeficient FAK-Y925F mutant were stably expressed into FAK -/- MEFs. ECM mediated FAK activation was induced by keeping cells in suspension (Susp) prior to plating them on fibronectin (FN). FAK activation was monitored by western blot as in Fig. 4. A representative experiment is shown in the left panel and the quantification is plotted in the right panel. For quantifications signals were normalized to FAK-WT signals observed in MEFs on FN. Error bars represent SD from three independent experiments. *p = 0.02; **p < 0.006 (unpaired Student t test)

We next asked which mechanism could be facilitating a switch of LD2 binding from FAT to vinculin. We therefore inspected the crystal structures of FAT bound to paxillin motifs LD2 and LD4, which at the high peptide concentrations used in the crystallographic studies were both found to bind to the H14 and H23 sites in FAT (Fig. 2A) [21]. Interestingly, Y925 in FAT, a known Src phosphorylation site [43], forms part of the H14 binding site. Modelling a phospho-tyrosine at the site suggests interference with LD binding (Fig. 5A), therefore indicating a possible mechanism for disfavoring LD2 binding to FAT. To test this in our binding assays we set out to generate FAT that is stoichiometrically phosphorylated on Y925 by performing kinase reactions with purified Src. Since Y925 in FAT was previously reported as an inefficient substrate for Src [3], we first performed pilot experiments under different conditions, using either the Src kinase domain (hSrc254-536) or a larger Src construct containing the SH3, SH2 and kinase domains (hSrc84-536) and quantified Y925 phosphorylation via an ELISA assay using a phosphospecific anti-FAK pY925 antibody. We found that FAT is only efficiently phosphorylated if heat-denatured prior to rapid cooling and addition of Src (Fig. 5B). When intending to scale this procedure up to obtain sufficient phosphorylated FAT for fluorescence anisotropy binding experiments, we observed heavy precipitation of FAT during the heating cycle, which did not redissolve after cooling and phosphorylation. We therefore decided instead to use a FAT-Y925E phospho-mimetic mutant, which has previously been used successfully [11]. In addition, we included a phosphodeficient FAT-Y925F mutant in the study. Fluorescence anisotropy experiments revealed that the Y925E and Y925F mutations had little effect on binding of LD1 and LD4, but significantly reduced binding of LD2 and LD2/4 (Fig. 5C-F). For LD2 the effect is modest, since as shown previously, LD2 retains considerable affinity to the H23 site (Fig. 2C). However, for the LD2/4 peptide, where the H23 site is occupied by LD4, the mutations significantly reduced the affinity to a range observed for single motif binding, suggesting release of LD2 from the H14 site. The effect of the Y925F mutation likely reflects a significant involvement of the Y925 hydroxyl group in LD2 binding (Fig. 2A). The result with FAT-Y925E supports the hypothesis from structural modelling that Y925 phosphorylation could be a mechanism for disfavoring LD2 binding to FAT, releasing it for interaction with vinculin, thereby promoting the FAK-vinculin linkage and FAK activation in response to force.

In order to validate these results, we tested whether Y925 phosphorylation is important for ECM mediated FAK activation in cells. For this, we stably expressed the Y925 phosphorylation deficient FAK mutant (FAK-Y925F) or the FAK-WT control in FAK -/- MEFs and determined FAK activation after plating these cells on fibronectin by monitoring phosphorylation of Y397 or Y576 and Y577. This revealed that upon cell adhesion to fibronectin FAK-Y925F expressing cells exhibited significantly reduced phosphorylation levels compared to FAK-WT expressing cells, indicating inefficient FAK activation in absence of Y925 phosphorylation (Fig. 5G). Taken together, biochemical and cellular results support a model where Y925 phosphorylation is a key step in ECM mediated FAK activation.

### LD2 attachment to vinculin is compatible with force transduction

**Fig. 6:**
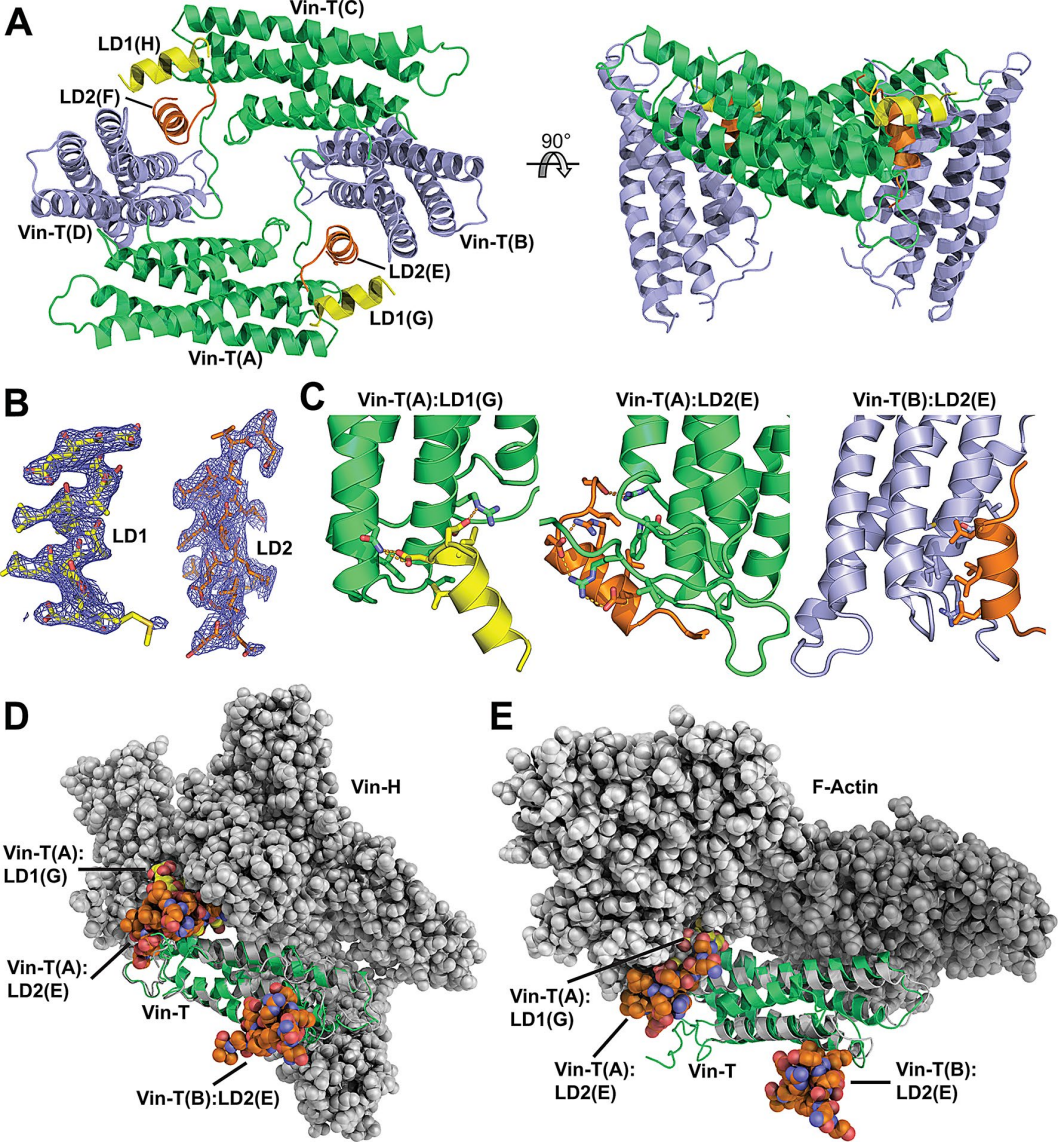
Crystal structure of vinculin tail (Vin-T) bound to paxillin peptides LD1 and LD2. **(A)** The asymmetric unit contains 4 Vin-T molecules (chains A and C colored in green; B and D in blue), 2 LD1 (chains G and H in yellow) and 2 LD2 peptides (chains E and F in orange). A 2-fold non-crystallographic symmetry axis (perpendicular to the plane of the left view) relates molecules of the same color to each other. Two perpendicular views are shown. **(B)** The LD1 and LD2 peptides are well defined by electron density maps. The blue mesh represents 2Fo-Fc electron density countered at 1σ. **(C)** Close-ups of Vin-T (chain A) interacting with LD1 (chain G) (left panel), Vin-T (A) interacting with LD2 (E) (middle panel) and Vin-T (B) interacting with LD2 (E) (right panel). **(D)** Superposition of Vin-T (green ribbon) bound to one LD1 (space-fill with carbons in yellow) and two LD2 peptides (space-fill with carbons in orange) with full-length vinculin (PDB: 1ST6) (grey, with the head domain, Vin-H, in space-fill and the tail domain as ribbon). The LD1 and two LD2 peptides are interacting with Vin-T via the three sites shown in (C) and the Vin-T and LD chain IDs for the interactions shown are indicated. **(E)** Superposition of Vin-T (green) bound to one LD1 and two LD2 peptides (coloring and labelling as in D) with the cryo-EM structure of Vin-T bound to F-actin (PDB: 3JBI) (grey, with Vin-T as ribbon and F-actin in space-fill). The position of the LD1 peptide bound to Vin-T is indicated, but mostly obstructed from the view

**Fig. 7:**
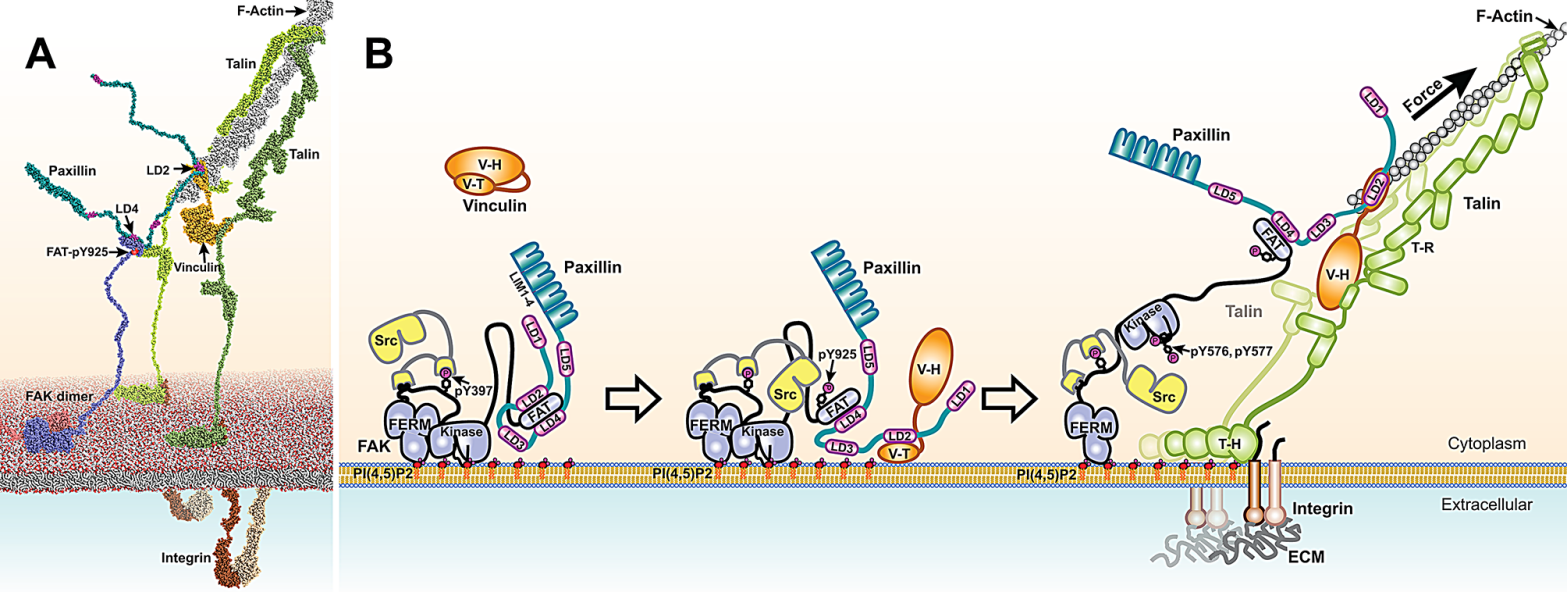
Model for phospho-regulated assembly of the FAK:paxillin:vinculin linkage and its role in FAK force-activation. **(A)** Atomic model of the FAK:paxillin:vinculin linkage and its connection to force transduction to integrins. Interactions are modelled based on the high-resolution structures of oligomeric FAK bound to membrane (only a dimer is shown for clarity, PDB: 6TY4), LD4 bound to FAT (PDB:1OW7), LD2 bound to Vin-T (reported here: Fig. 6C right panel), Vin-T bound to actin (PDB: 3JBI), the vinculin head domain bound to the talin VBS2 (PDB: 1U6H), talin head bound to PI(4,5)P2 (PDB: 6MFS), the talin head F3 lobe bound to β3-integrin tail (PDB: 2H7E) and the talin C-terminal dimerization helices (PDB: 2QDQ). FAK is colored blue with a modelled pY925 in red. A second FAK molecule is shown in red interacting via a dimerization interface on the membrane as observed by cryo-EM [2]. The C-terminal region including the FAT domain of this molecule is out of view to the left. Paxillin is colored in teal with LD motifs in magenta, vinculin is shown in bright orange, F-actin in grey, the talin dimer in green (with monomers in light or dark shade) and integrin with the alpha subunit in light brown and beta subunit in dark brown. See supplemental Fig. S3 for zoomed views of the interactions. **(B)** Schematic of proposed mechanism for FAK force activation in FAs. *Left*: FAK integrates into nascent FAs and interacts with PI(4,5)P_2_ lipids adopting a membrane bound conformation as observed by cryo-EM [2] with the kinase active site occluded by the membrane. PI(4,5)P_2_-induced FAK autophosphorylation on Y397 recruits the Src kinase. The FAK FAT domain interacts with the paxillin LD2 and LD4 motifs. *Center*: Recruited Src phosphorylates Y925 in the FAT domain via an unknown mechanism, resulting in release of paxillin LD2 from FAT. Vinculin is recruited and interacts via its tail domain (V-T) to paxillin LD2 and PI(4,5)P_2_ in the membrane. Vinculin interactions with PI(4,5)P_2_ and talin (latter not shown for clarity) induce an open conformation of vinculin with its head domain (V-H) detached from V-T. *Right*: Actin attaches to the talin rod domain (T-R) and V-T and contraction of stress fibers applies forces to FAs that stretch T-R and lift vinculin from the membrane, pulling along paxillin and the FAT domain of FAK. In turn, the FAK kinase domain is pulled off the membrane, thereby exposing the active site and activation loop residues Y576 and Y577 for phosphorylation by Src, resulting in FAK activation. Coloring is as in panel A. T-H: Talin head domain

The data presented above and existing structural data of FAT:paxillin [21] together provide a good structural model for paxillin attachment to FAT and Y925 phosphorylation mediated release of LD2. Yet, how LD2 interacts with vinculin and whether this interaction might be compatible with vinculin attachment to actin [30], and hence its role in force transduction, is unknown. We therefore set out to obtain structural information for LD2 binding to vinculin and performed crystallization experiments with Vin-T bound to LD2 and/or LD1 (the latter since we observed considerable affinity for LD1 to Vin-T; Fig. 1D). Interestingly, we only obtained crystals in presence of both, LD1 and LD2 peptides. Initial crystals were of poor quality, but diffraction quality crystals were obtained after using microseeds from initial crystals in a new round of crystallization screening (see methods). Diffraction of optimized crystals resulted in a crystal structure at a resolution of 2.55 Å (Tab.S3). In the final structure, the asymmetric unit contains four Vin-T molecules and each two LD1 and LD2 peptides, which are well defined in the electron density maps (Fig. 6A-B). Together, this assembly forms a circular arrangement that contains a non-crystallographic 2-fold symmetry. In order to test whether the LD1 and/or LD2 peptides could induce stable assembly of such a 4:2:2 arrangement in solution, we performed svAUC experiments with Vin-T in absence or presence of LD1, LD2 or both. At 20 µM, Vin-T alone forms a single species with a sedimentation coefficient of ~ 1.8 S, compatible with monomeric Vin-T (Fig. S2). At 100 µM approximately 20% of Vin-T converts to a form sedimenting with ~ 2.5 S, compatible with dimeric Vin-T. Neither the presence of LD1, LD2 or both peptides significantly changed the sedimentation behavior of Vin-T, suggesting that the multimeric assembly seen in the crystal structure does not form in solution at concentrations up to 100 µM. We therefore next asked which of the observed binary Vin-T:LD interactions (not resolved by svAUC) might reflect the interactions detected by fluorescence anisotropy (Figs. 1C-D). There are three different Vin-T:LD interactions observed in the crystal structure, one involving LD1 and two LD2 (Fig. 6C), each is present twice due to the non-crystallographic symmetry. Analysis with the PISA server [31] indicates that the most energetically favorable and most specific interaction (with significant hydrophobic character) is observed between LD2 and the Vin-T chains B and D (Table 1). This interaction involves the LD residues in LD2 and rigid residues within helices 2 and 3 (H2, H3) in Vin-T (Fig. 6C right panel). A second interface involving LD2 (but not the LD residues within the motif) is observed with Vin-T chains A and C and occurs in large part with loop regions in Vin-T (Fig. 6C middle panel). The LD2 interaction with helices H2 and H3 in Vin-T (chains B and D) is the only of the three interactions that is accessible unhindered in Vin-FL (Fig. 6D) as well as in Vin-T bound to actin (Fig. 6E). We therefore conclude that of the Vin-T:LD interactions observed in the crystal structure, only the LD2 interaction with helices H2 and H3 in Vin-T (Fig. 6C right panel) is compatible with a simultaneous role of vinculin in force transduction. Having obtained structural data for the LD2 interaction with Vin-T completes data required to reconstruct a full atomic model of FAK force activation via interactions that link FAK to paxillin (FAT:LD4, PDB: 1OW7 [21], paxillin to vinculin (Vin-T:LD2, presented in Fig. 6) and vinculin to actin (PDB: 3JBI [30]. We present such a reconstructed atomic model in the context of structural data known for the role of vinculin and talin in force transduction from actin to integrins (Fig. 7A, Fig. S3). This demonstrates that the existing structural data is fully compatible with the mechanistic model we propose for FAK force activation (Fig. 7B).

**Table 1:** Analysis of the LD:vinculin-tail (Vin-T) interactions observed in the crystal structure shown in Fig. 6 with PISA [31]. (1) solvation free energy gain. (2) measure of interface specificity with *P* < 0.5 indicating a specific interaction and *P* > 0.5 suggesting a non-specific interaction, possibly due to crystal packing

Protomer 1 (Chain ID)	Protomer 2 (Chain ID)	Interface Area (Å^2^)	Δ^i^G (kcal/mol)^(1)^	Δ^i^G (*P*-value) ^(2)^
Vin-T (A)	LD1 (G)	320.0	-3.0	0.541
Vin-T (C)	LD1 (H)	356.1	-2.4	0.561
Vin-T (A)	LD2 (E)	620.7	-1.7	0.670
Vin-T (C)	LD2 (F)	540.5	-3.2	0.605
Vin-T (B)	LD2 (E)	325.0	-6.0	0.161
Vin-T (D)	LD2 (F)	314.8	-5.8	0.194

## Discussion

Forces in FAs correlate with FAK activity [47, 50, 51, 57]. With the force transduction layer in FAs bridging the space between the signaling layer and actin [28], proteins within this layer are good candidates to communicate the FA force status to FAK. In support of this, the force transduction components talin and vinculin are required for a linear relationship between the force in individual FAs and phospho-FAK levels [57]. Intriguingly, structural data of FAK bound to the membrane revealed an inactive state with the kinase attached to the membrane [2], which pointed towards a possible force-activation mechanism by lifting the FAK kinase from the membrane via anchoring of the FAK C-terminus to force transduction components in FAs. The C-terminal FAT domain of FAK was indeed shown to interact with talin [33], yet since the interaction occurs with the talin head domain, which is thought to remain bound to integrins and the membrane during the FA life cycle, it is difficult to picture how the talin interaction could be involved in detaching the FAK kinase from the membrane. In contrast, vinculin initially interacts with PI(4,5)P2 [41] and then moves away from the membrane during FA maturation in a myosin II dependent manner [8], therefore fitting perfectly with a model of force induced removal of the FAK kinase from the membrane. However, vinculin is not known to directly interact with FAK. Here we present data supporting a model of a phospho-regulated tethering of FAK to vinculin via the FA adaptor paxillin (Fig. 7B). In this model, the FAT domain of FAK initially interacts tightly with the paxillin LD2 and LD4 motifs (Fig. 7B, left), followed by a Y925 phosphorylation mediated release of LD2 to establish a linkage to vinculin (Fig. 7B, middle). Forces mediated by actin are on the one hand transduced via talin and vinculin to integrins, providing traction for cell migration, and on the other hand are tethered via paxillin to FAK to activate signaling (Fig. 7B, right). We provide new structural information for the LD2:Vin-T interaction, which shows how simultaneous binding of Vin-T to LD2 and actin can ensure force transduction via vinculin, while being linked to FAK (Fig. 6E, Fig. S3D). Together with previous data, this allows a full structural reconstruction of our proposed model for FAK force activation. In Fig. 7A we show an atomic model of a state where the FAK kinase domain is still bound to the membrane while the C-terminal FAT domain is tethered via paxillin to Vin-T. The model incorporates high-resolution structures for all interfaces relevant to this model (see Fig. S3 for zoomed views of the interactions). The model shows a good spatial compatibility of simultaneous actin linkage to integrins and FAK. In addition to the structural compatibility, a number of cellular observations fit the proposed model: (i) Y925 phosphorylation is required for efficient ECM mediated FAK activation (Fig. 5G), (ii) detachment of the FAK kinase domain from the membrane by mutations at the membrane interaction site, as observed by cryo-EM, results in hyperphosphorylation of Y576/577 in the FAK kinase activation loop and increased cancer cell invasion [2], (iii) the force-FAK activity relationship requires vinculin [57], (iv) vinculin moves towards actin in a force dependent manner [8], potentially pulling along the FAK kinase via the paxillin tether, and (v) vinculin and paxillin adopt expected orientations in FAs with the vinculin C-terminus in average positioned above the N-terminus [8] and average paxillin N-termini positioned above their C-termini [28]. An intriguing point is that vinculin is not efficiently recruited to FAs in absence of force [20, 42], yet according to our model is required for FAK force activation. This indicates that initial force might build up via talin prior to efficient FAK activation, which according to Case et al. [8] very rapidly recruits vinculin to the membrane, allowing formation of the linkage to FAK.

Notably, in addition to LD2 and LD4, we also observe binding of LD1, both to FAT and Vin-T. LD1 interactions with FAT are of modest affinity and likely not important in the context of the FAT:paxillin:vinculin linkage, since it binds like LD4 with high specificity to the H23 site in FAT, yet with significantly lower affinity than LD4 (Fig. 2B and D). On the other hand, LD1 and LD2 can both bind simultaneously to Vin-T (Fig. 6). Interestingly, LD1 cannot bind efficiently to Vin-FL in the closed conformation, as shown by superposition with the Vin-FL structure (Fig. 6D) and confirmed by binding experiments with Vin-FL (Fig. 1C). It is therefore plausible that once vinculin localizes to the membrane, where its interactions with PI(4,5)P2 and talin induce an open vinculin conformation [4, 29], LD1 might help bringing Vin-T close to FAK even prior to LD2 release from the FAK-FAT domain. Regardless of the importance of LD1 in LD2 handover from FAT to Vin-T, LD1 will have to detach from Vin-T prior to actin attachment (Fig. 6E). LD1 is then free to interact with other FA proteins, as reported for alpha and beta parvin [38, 48] or p130 Cas [55].

Additional enhancing mechanisms, such as via LD1, might indeed be important in light of the low efficiency of Y925 phosphorylation (Fig. 5B) [3], which is in fact also observed in cells [44]. Whether such mechanisms exist is currently not clear, but interestingly tension in the H1/H2 linker is shown to promote H1 release [3, 26]. A plausible mechanism is that although Y925 phosphorylation is inefficient initially, which might prevent uncontrolled FAK activation, once phosphorylated and force applied to FAK, the force could promote H1 release from the FAT 4-helix bundle, thereby ensuring efficient maintenance of Y925 phosphorylation considering the cellular kinase/phosphatase balance. This could ensure that FAK is kept sufficiently long under force for kinase release from the membrane.

As mentioned above, the FAT interaction with the talin head domain located at the membrane [33] has unlikely a direct role in force transmission to FAK. Rather, the talin interaction with FAT and vinculin could point to an important mediator and/or adaptor function for talin during early FA assembly on the membrane, which could explain its requirement for establishing a force-activity relationship for FAK [57]. An important role for talin in assembling FA components on the membrane is further supported by recent observations showing that PI(4,5)P2 and vinculin interactions of talin favor the formation of 2D condensates on the membrane [37]. Such condensates would likely also promote the inclusion of other FA components, such as FAK via its interactions with talin and PI(4,5)P2, thereby assembling all the players for both, productive force transduction to integrins and activation of FA signals.

## Materials and methods

### Protein expression and purification

Expression plasmids for FAT (hFAK904-1052 WT and mutants H14, H23 and H14-23), Vin-FL (hVinculin1-1066) and Vin-T (hVinculin891-1066) were ordered from GenScript in the pET28(+) vector, adding a TEV cleavable N-terminal 6xHis tag to the expressed proteins. Mutant FAT-H14 contains the mutations I936A, H1025A, K1032A; FAT-H23 contains the mutations K955A, R962A, I998A and FAT-14-23 contains the combined mutations I936A, K955A, R962A, I998A, H1025A, K1032A. All plasmids were introduced into *E.coli* strain BL21(DE3) for expression. Cells were grown in LB media, induced with 1 mM IPTG at an OD (600 nm) of 0.6 and grown over night at 20 °C. Cells were pelleted at 6000 rpm and resuspended in 20 mM Tris pH8, 200 mM NaCl, 5% Glycerol, 10 mM imidazole, 2mM TCEP, 1mM PMSF supplemented with cOmplete protein inhibitor cocktail (Roche). Cells were lysed by sonication and insoluble components removed by centrifugation at 40 K for 1 h at 4 °C. Soluble lysates were cleared through 0.45 μm filters and loaded onto His-Trap affinity columns (Cytiva), washed with step gradients up to 30 mM imidazole and eluted with a gradient up to 500 mM imidazole. Peak-fractions were digested over night with TEV protease (molar ratio ~ 1: 20) at 4 °C. Proteins were diluted to 50 mM NaCl and loaded onto a Source 15Q column, washed with 20 mM Tris pH8, 50 mM NaCl, 5% Glycerol, 1mM TCEP and eluted with a gradient up to 1 M NaCl. Peak fractions were mixed with 1 ml of Ni-NTA beads to remove TEV and uncleaved protein, adding 10 mM imidazole to prevent non-specific binding. The flow-through was concentrated, loaded onto a Superdex200 16/60 column and eluted in 20 mM Tris pH8, 200 mM NaCl, 5% Glycerol, 2mM TCEP. Peak fractions were concentrated, aliquots flash frozen in LN2 and stored at -80 °C.

### Fluorescence anisotropy

Binding curves were determined in a total of 20 µl in black 384 well plates, by titrating protein concentrations (typically up to 50 µM) at a fixed concentration of 25 nM FITC labelled LD peptides (Apeptide Co. Ltd) in 20 mM Tris pH7.5, 150 mM NaCl, 1mM TCEP. Anisotropy measurements were preformed at 22 °C on a Spark^®^ Multimode microplate reader (Tecan) with polarizers, at an excitation wavelength of 485 nm and an emission wavelength of 535 nm (20 nm bandwidth). K_D_ values were extracted by fitting a one-site binding model using the GraphPad Prism software, using the equation: Y = B_max_*X/(K_D_ + X), where Y is the measured anisotropy, X the protein concentration and B_max_ the maximal anisotropy value at binding saturation.

### Sedimentation velocity analytical Ultracentrifugation(svAUC)

For svAUC experiments, FAT and Vin-T proteins at 30 µM were incubated with 10 µM LD2/4 peptide in 20 mM Tris pH 7.5, 200 mM NaCl, 5% Glycerol and 1 mM TCEP. svAUC was performed at 20 °C at 48,000 rpm (Vin-T + LD1 + LD2, Fig. S2) or 58,000 rpm (FAT + LD2/4 + Vin-T; Fig. 3) in a XL-I analytical ultracentrifuge (Beckman-Coulter Inc.) equipped with both UV-VIS absorbance and Raleigh interference detection systems, using an An-50Ti rotor, and Epon-charcoal standard double-sector centerpieces (optical pathlength 12 mm or 4 mm for measurements at 100 µM shown in Fig. S2). Sedimentation profiles were recorded at 280 nm. Differential sedimentation coefficient distributions were calculated by least-squares boundary modelling of sedimentation velocity data using the continuous distribution c(s) Lamm equation model as implemented by SEDFIT [46]. These s values were corrected to standard conditions (water, 20 °C, and infinite dilution) [22] using the program SEDNTERP [32] to get the corresponding standard s values (s20,w).

### GST-pulldown assay

30 µM GST-Vin-T, 10 µM LD2/4 and 30 µM His-FAT proteins were incubated with glutathione sepharose beads for 1 h, centrifuged for 1 min at 3.3 rcf and washed 3x with 20 mM Tris pH 7.5, 150 mM NaCl, 0.1% Nonidet P40. Proteins co-sedimented with beads were resuspended in SDS Laemmli buffer, heated to 96 °C for 5 min and subjected to SDS-PAGE and western blotting. His-FAT proteins (WT or mutants) were detected using a primary mouse anti-His antibody (Proteintech) and a secondary HRP conjugated goat anti-mouse antibody (Merck).

### FAT-Y925 phosphorylation assay

Phosphorylation reactions were performed with 0.6 µM FAT, 0.2 µM Src (either Src kinase domain, Src254-536, or Src SH3-SH2-kinase, Src84-536, as indicated), 0.8 mM ATP and 4 mM MgCl_2_, incubated at 25 °C and stopped at indicated time points with 30 mM EDTA (final). For preheated samples, FAT was heat denatured at 95 °C for 10 min followed by rapid cooling to 25 °C prior to adding the other components. FAT pY925 levels were detected using an ELISA protocol. In brief, samples were diluted 10-fold with 0.1 M bicarbonate/carbonate buffer pH 9.6 and transferred to Maxisorp ELISA microtiter plates (Nunc). Plates were coated, blocked with 4% BSA in PBS, incubated with primary (1:2000 anti-FAK pY925 antibody, Cell Signaling Technologies) and secondary (1:1000 goat anti-rabbit-Ig conjugated with HRP) antibodies and developed with TMP for 2 min. Colorimetric reactions were stopped with 0.18 M sulfuric acid and absorption read at 450 nm using a Varioskan plate reader (Thermo Fisher). Each data point was performed in two independent reactions, each determined by ELISA in duplicates (total *n* = 4 per sample).

### FAK activation in cells

Lentiviruses encoding for GFP-FAK-WT, GFP-FAK-Y925F, GFP-FAK-H23 or GFP alone were used to stably reconstitute FAK -/- MEFs. To obtain equal expression levels, cells were sorted at equal GFP fluorescence intensity. To determine the activation of FAK-WT, FAK-Y925F and FAK-H23 in response to integrin activation, cells were starved in DMEM containing 0.5% fetal bovine serum for 16 h, trypsinized and held in suspension in DMEM with 0.5% BSA for 45 min at 37 °C. 7 × 10^5^ cells in 1 ml of medium were plated on fibronectin (FN)-coated plates, and incubated for 15 min in the incubator. FN coating was carried out overnight at 10 ug/ml in PBS and blocked with 1% BSA for 30 min before washing with PBS. Cells were lysed in 70 ul of lysis buffer (50 mM Hepes, pH 7.4, 150 mM NaCl, 1% Triton X-100, 1% sodium deoxycholate, 0.1% SDS, 100 mM NaF, 1.5 mM MgCl_2_, 1 mM EDTA, 10 mM sodium pyrophosphate, 1 mM sodium orthovanadate, 10% glycerol, 1 mg/ml Leupeptin, 1 mg/ml Aprotinin, 1 mM PMSF and cOmplete protease inhibitor cocktail (Roche)) and loaded onto an SDS-PAGE gel. Phosphorylation on Y397 and Y576/Y577 was detected by western blot using phospho-specific antibodies (Cell Signaling Technology), HRP conjugated secondary antibody and chemiluminescence detection (Fisher Scientific). Data was obtained from three independent experiments and quantified using the Image J Software.

### Crystallization and data collection

For crystallization experiments Vin-T at 6.1 mg/ml was incubated with 180 µM LD1 and/or 90 µM LD2 (lower concentration due to solubility) for 15 min on ice. Initial crystals were obtained for Vin-T in presence of LD1 and LD2 using the vapor diffusion method and mixing Vin-T:LD complexes with equal volume of precipitation solution containing 150–175 mM (NH4)_2_SO_4_, 19–20% PEG 1500, 0.1 M Na Acetate pH 5 and 10 mM TCEP. Crystals appeared as bunches of needles unsuitable for diffraction and were therefore used to produce microseed stocks for new crystallization screenings. Diffraction grade crystals were finally grown by mixing samples with equal volume of precipitant solution containing 1.26 M (NH4)_2_SO_4_, 0.2 M NaCl, 0.1 M Na Acetate pH4.5. Crystals were cryoprotected with precipitant solution containing additionally 25% ethylene glycol and 200 µM LD1 and 100 µM LD2 peptides and flash frozen in liquid nitrogen. Diffraction data was collected at the XALOC-BL13 beamline of the ALBA synchrotron (Barcelona, Spain) at 100 K using a PILATUS 6 M detector.

### Structure determination and refinement

Diffraction data was indexed and integrated with XDS [25] and scaled with AIMLESS [15], applying a high resolution cut-off of 2.55 Å. Crystals were determined to belong to the C2 space group with unit cell parameters a = 177.38 Å, b = 70.56 Å, c = 117.37 Å and angles α = 90°, β = 131.25° and γ = 90°. An initial model was generated by molecular replacement using PHASER [39] and the Vin-T coordinates (PDB = 1QKR) as search probe, yielding 4 clear solutions. Refinement and building of the LD1 and LD2 peptides was performed by iterative cycles of restrained refinement, including TLS cycles, with REFMAC5 [40] and manual building guided by electron density maps, using COOT [12, 13]. The final model contains 4 Vin-T molecules and each 2 LD1 and LD2 peptides and contains a 2-fold non-crystallographic symmetry. Final R-factors are 22.5/25.9 (work/free) (for full crystallographic table, see supplementary Table S3).

### Modelling

The atomic model presented in Fig. 7A was generated using the following PDB coordinates: Entry 6TY4 was used for dimeric membraned bound FERM and kinase domains of FAK, 1OW7 for the FAT domain of FAK bound to paxillin LD4, 3JBI for Vin-T bound to actin, 1TR2 for the full vinculin head domain, 1U6H for the vinculin head interaction with talin vinculin binding site 2 (VBS2), 6MFS for the talin head domain bound to the PI(4,5)P2 membrane lipid, 1MIZ and 2H7E for the talin head interaction with the β3-integrin tail, 6R9T for talin rod helical bundles R1-R2 and R4-R12 which were rearranged into an extended conformation, 2JSW for the talin R13 actin binding domain and 2QDQ for the talin C-terminal dimerization helix. 8GCD and 3T3M were used to model an open conformation of extracellular and transmembrane regions of αIIbβ3 integrin. For the Vin-T:LD2 interaction we used Vin-T chain B bound to LD2 chain E from the structure presented in this study. Y925 in the FAK FAT domain was replaced by pY925 (3 letter code PTR). Unstructured and/or missing regions in PDB files were modelled from full-length alpha-fold predictions and rearranged to position interacting partners. The full model was assembled and rearranged in COOT [12, 13] applying geometry regularization to all modelled regions not defined by high-resolution structures.

### Molecular dynamics simulations

The FAT:paxillin LD2-LD4 complexes were modelled with LD2 and LD4 interacting to the H14 and H23 site in FAK respectively, according to crystallographic structures [21]. Non interacting native regions of paxillin were obtained from alpha-Fold and rearranged and geometry regularized in COOT, as was the non-native linker in LD2/4. MD simulations were performed using GROMACS software [1] v2021.5, employing OPLS force field [24] and TIP3 water model. For each simulated system, the FAT:paxillin LD2-LD4 complex was placed at the center of a cubic periodic boundary box filled with water molecules. All ionizable residues in the protein were kept in their most probable protonation state at neutral pH. Sodium ions were added to neutralize the entire system and, additionally, both sodium and chloride ions were added to maintain 0.15 M NaCl conditions. Before the molecular dynamic simulations, an energy minimization step was performed using the steepest descent algorithm. Following the energy minimization, the minimized system was subjected to equilibration in two phases: NVT (constant Number of particles, Volume, and Temperature) and NPT (constant Number of particles, Pressure, and Temperature). The production run was carried out at a temperature of 300 K and a pressure of 1 atmosphere for 100 ns. The temperature and pressure were maintained using a velocity rescaling thermostat and Parrinello-Rahman barostat [6]. All bonds connected to hydrogen atoms were constrained using the LINCS algorithm [19], and the time step was set to 2 fs. Long-range Coulomb interactions were calculated using the smooth particle mesh Ewald method [14], with a grid spacing of 0.16 nm. The real space cut-off for both the Coulomb and van der Waals interactions was 1.2 nm. During this production run, the dynamic behavior and stability of each complex were analyzed using various parameters. These analyses were performed using built-in tools in the Gromacs software.

## Electronic supplementary material

Below is the link to the electronic supplementary material.

Supplementary Material 1

## Data Availability

The structure of Vin-T bound to paxillin LD1 and LD2 is deposited in the protein data bank (PDB) under accession code 9QWO.
