# Supplementary material for: Phospho-regulated tethering of focal adhesion kinase to vinculin links force transduction to focal adhesion signaling
Source: Cell Commun Signal. 2025 Apr 21;23:190. doi: 10.1186/s12964-025-02201-3 (PMC12013189; doi:10.1186/s12964-025-02201-3)
Supplement: Supplementary file 1 — Supplementary Material 1 [file 12964_2025_2201_MOESM1_ESM.pdf]

Supplemental information

Supplementary Table S1: Paxillin peptides used in the study.

LD1	MDDLDALLADLES
LD2	SNLSELDRLLLELNAVQHN
LD3	SVESLLDELES
LD4	SATRELDELMASLSD
LD5	SQLDSMLGSLQSD
LD2/4	SNLSELDRLLLELNAVQHN*GSGSGGSGGSGGSGGSGGSGGSGGSGG*SATRELDELMASLSD

For anisotropy experiments peptides were labelled N-terminally with FITC. The non-native linker region connecting LD2 and LD4 sequences in peptide LD2/4 is in italic.

Supplementary Table S2. Integration of peaks from AUC

**Sample**	**c(Peak 1) [~ 1 s(S)]**	**c(Peak 2) [1-2 s(S)]**	**c(Peak 3) [>2 s(S)]**	**c(total)**
FAT-WT		0.30	0.05	0.35
FAT-H14		0.37		0.37
FAT-H23		0.36		0.36
Vin-T		0.52		0.52
FAT-WT+LD2/4	0.06	0.37	0.15	0.58
FAT-H14+LD2/4	0.11	0.45	0.05	0.61
FAT-H23+LD2/4	0.11	0.45	0.03	0.59
Vin-T+LD2/4	0.18	0.59		0.77
FAT-WT+LD2/4+Vin-T		0.68	0.40	1.08
FAT-H14+LD2/4+Vin-T		0.57	0.52	1.09
FAT-H23+LD2/4+Vin-T	0.08	0.80	0.30	1.18

c-values are calculated by integration of peaks shown in Fig.3.

Supplementary Table S3. Crystallographic data collection and refinement statistics

	Vinculin-T+LD1+LD2
**Data collection**	
X-ray source	ALBA, BL13-XALOC
Wavelength (Å)	0.97926
Space group	C2
Cell dimensions	
a, b, c (Å)	177.38, 70.56, 117.37
α, β, γ (°)	90, 131.25, 90
Resolution (Å)	44.83-2.55 (2.69-2.55)*
Total reflections	109790 (16149)
Multiplicity	3.1 (3.1)
Unique reflections	35621 (5168)
Completeness (%)	99.6 (99.9)
R_{merge} (%)	10.1 (85.6)
R_{meas} (%)	12.4 (103.5)
R_{pim} (%)	6.9 (57.5)
CC(1/2)	99.5 (61.1)
$I / \sigma I$	7.3 (1.4)
**Refinement**	
Resolution (Å)	44.87-2.54
Reflections (total/test set)	34219/1677
R_{work} / R_{free} (%)	22.5/25.9
No. atoms	5926
Protein	5745
Solvent	101
Other	80
R.m.s. deviations	
Bond lengths (Å)	0.005
Bond angles (°)	0.996
mean B value (Å^2)	70.1

*Values in parentheses are for highest-resolution shell.

Supplementary Figures:

Figure S1:

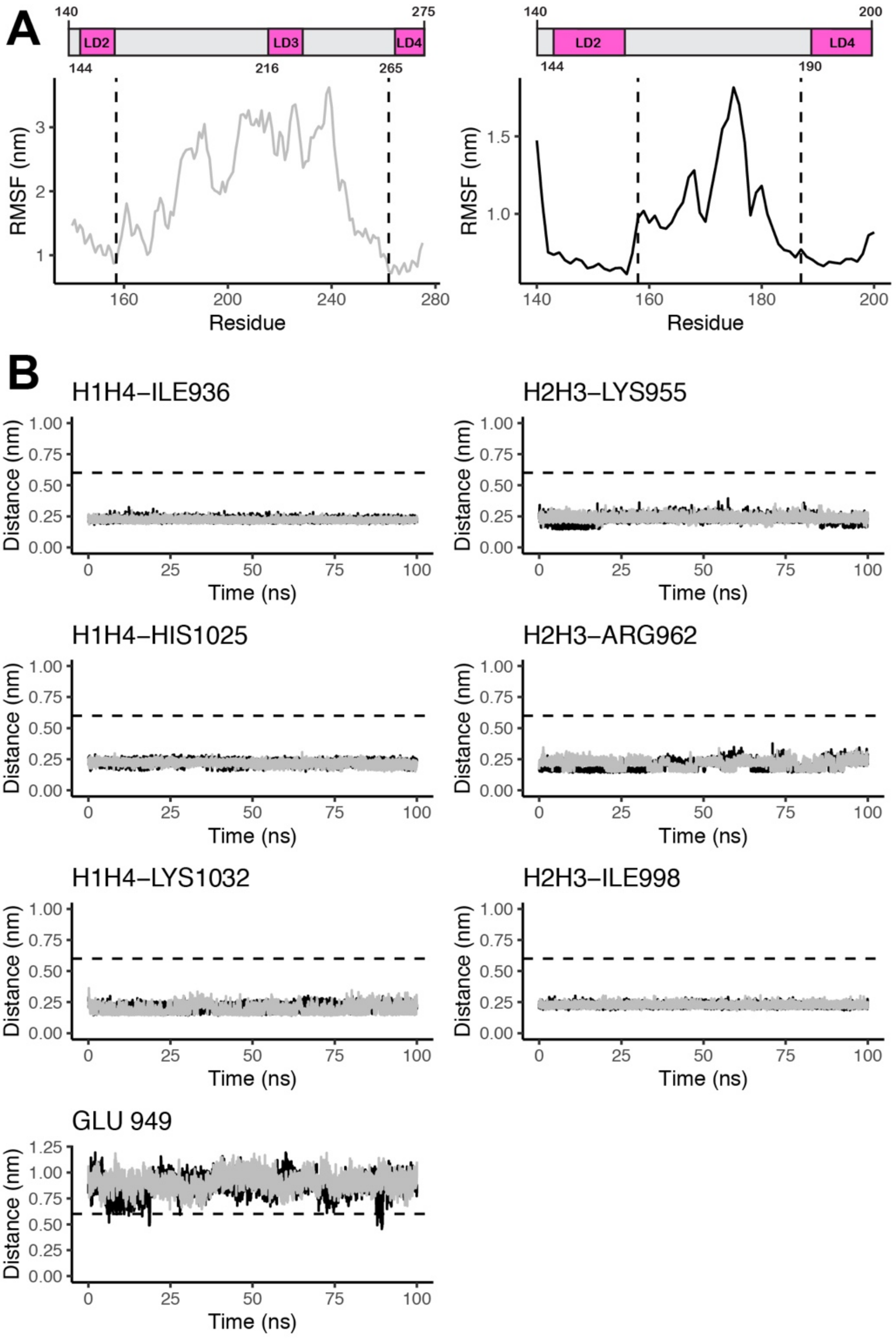

Molecular dynamics simulations of paxillin LD2 and LD4 bound to FAT. **(A)** Root mean square fluctuations (RMSF) of the interacting native paxillin LD2-LD4 region (left) or the LD2/4 peptide used in this study (right), bound via LD2 to the H1H4 and via LD4 to H2H3 binding site in FAT, are plotted for Cα atoms **(B)** Minimal FAT-paxillin distances for interacting residues within the LD2:H1H4 (ILE936, HIS1025, LYS1032) or the LD4:H2H3 (LYS955, ARG962, ILE998) binding sites or for a non-interacting residue (GLU949) are plotted throughout the simulation for the FAT interaction with the native LD2-LD4 region (grey) or the LD2/4 peptide used in the study (black). In both cases, the LD2 and LD4 interactions remain stable throughout the simulation, well within interaction distance.

Figure S2:

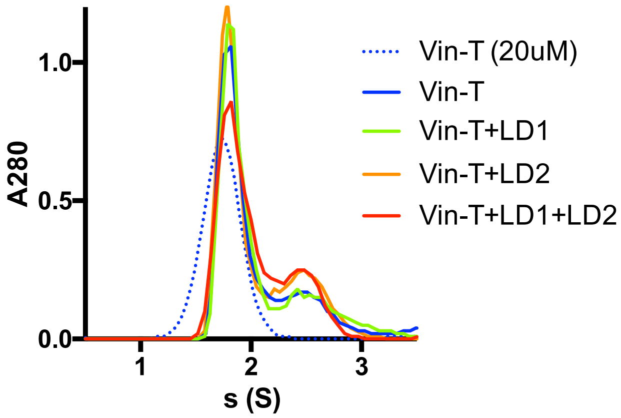

Sedimentation velocity analytical ultracentrifugation (svAUC) analysis of vinculin tali (Vin-T) alone or bound to LD1 and/or LD2. Measurements are performed at 100 uM Vin-T and LD1/LD2 unless indicated. At 20 uM Vin-T appears as a homogenous monomer, while at 100 uM approximately 20% of Vin-T forms a dimer. Formation of Vin-T dimers or higher oligomers are not significantly affected by the presence of LD1 and/or LD2.

Figure S3:

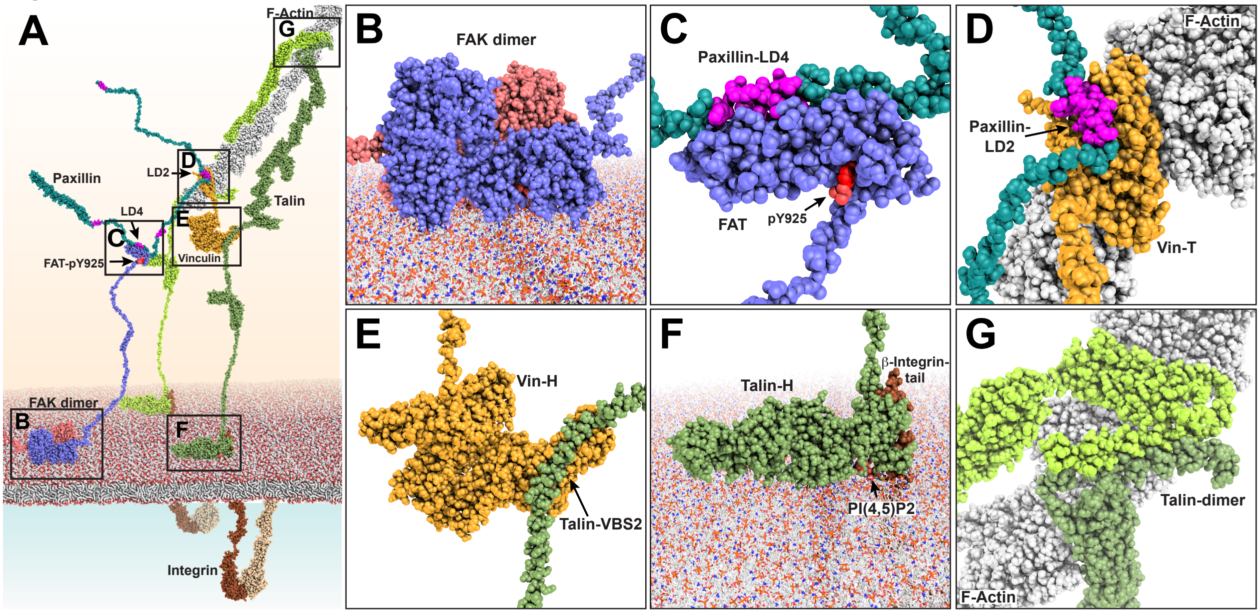

Atomic model of FAK force activation. **(A)** Atomic model as in Fig.7A. Boxed are regions based on high-resolution structures, which are shown enlarged in panels B-G. Coloring as in Fig.7A. **(B)** Symmetric FAK dimer bound via FERM and kinase domains to the membrane with the kinase active site facing the membrane as in PDB 6TY4 (Acebrón *et al*, 2020). **(C)** Paxillin LD4 bound to the H23 site in FAT, as in 1OW7 (Hoellerer *et al*, 2003). Y925 (red) is modelled in a phosphorylated state resulting in an unbound H14 site. **(D)** Paxillin LD2 bound to Vin-T as reported in this study (Fig.6C right panel) and Vin-T bound to actin as in 3JBI (Kim *et al*, 2016). **(E)** Vin-H bound to talin VBS2 as in 1U6H (Fillingham *et al*, 2005). **(F)** The talin head (H) domain is bound to the membrane lipid PI(4,5)P2 as in 6MFS (Chinthalapudi *et al*, 2018) and to the β3-integrin tail as in 1MIZ (Garcia-Alvarez *et al*, 2003) and 2H7E (Wegener *et al*, 2007). **(G)** Talin C-terminal helices are dimerized as in 2QDQ (Gingras *et al*, 2008) and the talin rod domain R13 modelled bound to actin, loosely based on the low resolution structure in (Gingras *et al.*, 2008).

References

Acebrón I, Righetto RD, Schoenherr C, de Buhr S, Redondo P, Culley J, Rodriguez CF, Daday C, Biyani N, Llorca O *et al* (2020) Structural basis of Focal Adhesion Kinase activation on lipid membranes. *EMBO J* 39: e104743

Chinthalapudi K, Rangarajan ES, Izard T (2018) The interaction of talin with the cell membrane is essential for integrin activation and focal adhesion formation. *Proc Natl Acad Sci U S A* 115: 10339-10344

Fillingham I, Gingras AR, Papagrigoriou E, Patel B, Emsley J, Critchley DR, Roberts GC, Barsukov IL (2005) A vinculin binding domain from the talin rod unfolds to form a complex with the vinculin head. *Structure* 13: 65-74

Garcia-Alvarez B, de Pereda JM, Calderwood DA, Ulmer TS, Critchley D, Campbell ID, Ginsberg MH, Liddington RC (2003) Structural determinants of integrin recognition by talin. *Mol Cell* 11: 49-58

Gingras AR, Bate N, Goult BT, Hazelwood L, Canestrelli I, Grossmann JG, Liu H, Putz NS, Roberts GC, Volkmann N *et al* (2008) The structure of the C-terminal actin-binding domain of talin. *EMBO J* 27: 458-469

Hoellerer MK, Noble ME, Labesse G, Campbell ID, Werner JM, Arold ST (2003) Molecular recognition of paxillin LD motifs by the focal adhesion targeting domain. *Structure* 11: 1207-1217

Kim LY, Thompson PM, Lee HT, Pershad M, Campbell SL, Alushin GM (2016) The Structural Basis of Actin Organization by Vinculin and Metavinculin. *J Mol Biol* 428: 10-25

Wegener KL, Partridge AW, Han J, Pickford AR, Liddington RC, Ginsberg MH, Campbell ID (2007) Structural basis of integrin activation by talin. *Cell* 128: 171-182
